# Moderating effect of social capital on income and oral health-related quality of life during pregnancy

**DOI:** 10.1590/1807-3107bor-2025.vol39.100

**Published:** 2025-10-20

**Authors:** Sabrina Cabral PACHECO, Gabriela de ARAUJO, Maiara Carvalho SEGATTO, Jessica Klockner KNORST, Fernanda TOMAZONI, Bruno EMMANUELLI

**Affiliations:** (a) Universidade Federal de Santa Maria - UFSM, School of Dentistry, Department of Stomatology, Santa Maria, RS, Brazil.

**Keywords:** Quality of Life, Oral Health, Social Capital, Pregnant People

## Abstract

This cross-sectional study aimed to evaluate the moderating role of social capital in the relationship between income and oral health-related quality of life (OHRQoL) among pregnant women. The study was conducted using a random sample of pregnant women registered with the public health system in southern Brazil. Women with sensory or cognitive impairments, non-Brazilian nationality, or high-risk pregnancies were excluded. The Oral Health Impact Profile (OHIP-14) was used to assess OHRQoL. Social capital was evaluated through “perceived social support” and “pregnancy group attendance.” The monthly household income was assessed in Brazilian Reais and categorized as either high (≥ 1,000 BRL) or low (< 1,000 BRL). The moderating role of social capital on the relationship between income and OHRQoL was tested using multilevel Poisson regression analysis. The results are presented as rate ratio (RR) with 95% confidence intervals (CI). A total of 520 pregnant women were assessed, with a response rate of 93%. Approximately 25.6% had low income. Both social capital variables demonstrated a moderating effect on the relationship between income and OHRQoL. Among pregnant women with a low household income, those without social support and those who did not attend pregnancy groups had 44% (RR = 1.44, 95%CI: 1.22–1.72) and 22% (RR = 1.22, 95%CI: 1.05–1.42) higher overall OHIP-14 scores, respectively, compared to their counterparts. This means that social support and group attendance substantially mitigate the negative effects of low income on OHRQoL. Our findings highlight the potential role of social capital promotion in this population.

## Introduction

Oral health-related quality of life (OHRQoL) is a multidimensional concept that encompasses the functional, psychological, and social aspects of oral health, along with the experience of pain and discomfort, and their impact on an individual’s well-being and daily life.^1,2^ The role of social determinants of health on OHRQoL has been well-documented in the literature.^3-5^ Among these factors, socioeconomic aspects stand out, particularly household income, one of the most commonly used income indicators in health research.^6^ In general, lower income has been associated with poorer clinical oral health outcomes and reduced OHRQoL.^3,7,8^ In this context, individuals from low socioeconomic backgrounds face multiple risk factors, including limited access to dental care, poorer oral health habits, and higher stress levels, all of which contribute to poorer general and oral health.^9-11^

In addition to socioeconomic factors, social capital has emerged as a key social determinant of health.^12^ Social capital refers to the resources acquired through participation in various social networks, as well as the interpersonal trust fostered within these connections.^13-15^ Studies have shown that low levels of social capital are linked to poorer oral health outcomes, including dental caries and OHRQoL, across different populations.^16-18^ Studies involving pregnant women have shown that low individual social capital is associated with unhealthy behaviors (e.g., smoking, drinking, and poor nutritional status), as well as a poorer diet and lower OHRQoL.^16,19^

Given the significant influence of social capital on oral health, it is hypothesized that its levels may either mitigate or amplify the impact of socioeconomic inequalities on oral health, including during pregnancy. Some studies have explored the mediating and moderating roles of social capital in the relationship between various social determinants and individuals’ oral health.^20-22^ To the best of our knowledge, however, no study has investigated the moderating role of social capital on OHRQoL in pregnant women. Pregnancy is a critical period marked by physiological and behavioral changes, which can increase vulnerability to oral health issues.^23,24^

Understanding the interplay among these factors is crucial for developing effective oral health promotion strategies and future interventions tailored to this population This study aimed to evaluate the moderating role of social capital in the association between income and OHRQoL during pregnancy. Our conceptual hypothesis is that lower social capital levels may intensify the impact of low income on OHRQoL.

## Methods

### Ethical concerns

This study was approved by the of Research Ethics Committee of the Federal University of Santa Maria, Brazil (CAAE process no. 54969222.9.0000.5346). All participants were informed about the objectives of the study and signed an informed consent form.

### Study design and sample

A survey was conducted with a representative sample of pregnant women who were users of public primary health centers in Santa Maria, a town in southern Brazil. The study was carried out from May 2022 to November 2022, and according to official data, the city had an estimated population of 271,633 inhabitants.^25^ At the start of the study, data from the Local Department of Health indicated that more than 1,380 pregnant women were registered at public primary health centers. In the Brazilian National Healthcare System (Sistema Único de Saúde, SUS),^26^ all pregnant women are entitled to comprehensive prenatal care. According to official local data, 67% of pregnant women registered at public health centers received dental prenatal care in 2022.^27^

All pregnant women regularly registered at each of the 25 public primary health centers providing prenatal care were considered eligible to participate in the study. A cluster sampling procedure was then implemented. The selection process accounted for the weight of each data collection point (public primary health center), which was calculated as the ratio between the number of pregnant women registered at a specific health center and the total number of pregnant women enrolled in the public service at the start of the study. For recruitment, the researchers scheduled visits to the public primary health centers on days designated for medical appointments for pregnant women. During these visits, pregnant women were invited to participate in the study.

The minimum sample size was estimated using the GPower Software version 3.1. Sample size calculation was based on the mean differences test, with the following parameters: a 95% confidence interval, 80% power, a 1:1 ratio of exposed to non-exposed individuals, and an effect size of 0.2. The design effect was taken into account, and the number obtained was multiplied by 1.2. Additionally, 10% was added to account for possible refusals, resulting in a minimum sample size of 519 pregnant women. Women with vision or hearing problems, cognitive impairments, those who were not Brazilian, or those at-risk during pregnancy were excluded from the study.

### Data collection

Data collection was conducted using validated methods^27-30^ by a team of five dentists who served as both examiners and interviewers. Non-clinical variables were collected through structured questionnaires administered via face-to-face interviews. Clinical variables were assessed in dental chairs using a plane dental mirror and periodontal probles (CPI; “ball point”) under natural light. The team was previosly trained and calibrated. This process consisted of theoretical classes (3 hours), image-based training (1 hour), and clinical evaluations (4 hours).^29^ The intra-examiner kappa coefficients were above 0.80, while inter-examiner reproducibility ranged from 0.76 to 0.88.

OHRQoL was evaluated by the Brazilian version of the Oral Health Impact Profile (OHIP-14).^28^ This questionnaire comprises seven dimensions: functional limitations, physical pain, psychological discomfort, physical disability, psychological disability, social disability, and handicap. Each dimension has two items with scores assigned on a 5-point Likert scale, ranging from 0 to 4 points: (0) never, (1) hardly ever, (2) occasionally, (3) fairly often, and (4) very often. The OHIP-14 total scores range from 0 to 56 points, with higher scores indicating poorer OHRQoL.

Our main predictor, household monthly income in the previous month, was collected in Brazilian Reais (R$) as a continuous variable and later dichotomized. ‘High’ income included the upper three quartiles (Q2, Q3, and Q4), while ‘Low’ income referred to the poorest quartile (Q1), representing households earning less than R$1,000. For reference, one US dollar (USD $1.00) is approximately equivalent to R$5.00.

Social capital, the moderating factor of interest, was assessed using two questions: (I) ‘If something unfortunate happened to you, would anyone help you in this situation?’—used as a proxy for social support, representing cognitive social capital; and (II) ‘Do you attend pregnancy groups?’— used as a proxy for social networks.^14,15^ For the first question, pregnant women were categorized as having or not having social support, while the second question had ‘yes’ or ‘no’ response options. Similar questions have been used in previous studies as proxies for individual social capital.^21,30^

Additional covariates were evaluated and considered as potential confounding factors. Demographic characteristics included age (in years) and skin color (white or black/brown).^25^The duration of pregnancy was recorded in weeks and subsequently categorized into the first, second, and third trimesters. The use of dental services in the past 12 months was assessed as a behavioral variable. For analytical purposes, it was categorized into ‘>1 time per year’ and ‘< 1 time per year’.^29^Dental caries was assessed through the decayed, missing and filled teeth (DMFT) index.^29^ The presence of the D component of the DMFT index was considered as untreated dental caries. This variable was dichotomized into ‘Present’ (D component ≥ 1) and ‘Absent’ (D component = 0).^29^

### Statistical analyses

Data analysis was conducted using STATA 14.0 statistical software (StataCorp. 2014. Stata Statistical Software: Release 14.0. Stata Corp L, College Station, USA). A descriptive analysis of sample characteristics was presented. The outcome (OHRQoL) was evaluated using the overall OHIP-14 scores.

Multilevel Poisson regression analyses were conducted to assess the moderating effect of social capital on the relationship between household income and OHRQoL. In the multilevel structure, pregnant women (1st level) were nested in public primary health centers (2nd level). All statistical models were guided by a theoretical framework presented in Figure, adapted from WHO,^29^ in which possible confounders were considered. Interaction relationships were considered for the two main potential moderators — social support and attendance at a prenatal group — using the following categorical classifications for social support: 0 = High income × with social support; 1 = High income × without social support; 2 = Low income × with social support; and 3 = Low income × without social support. The same categories were applied to the model that considered attendance at a prenatal group: 0 = High income × attended prenatal group; 1 = High income × did not attend prenatal group; 2 = Low income × attended prenatal group; and 3 = Low income × did not attend prenatal group. Tests on the multiplicative interaction scale were used to assess effect modification.^31^The results are presented in rate ratio (RR) and 95% confidence interval (95%CI).

**Figure:**
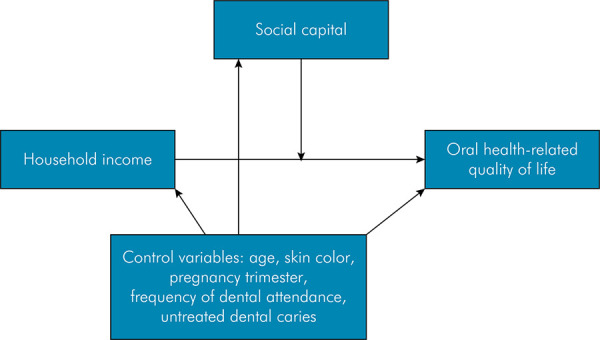
Logic map illustrating the moderating effects and confounding factors.

The simple slope test was also conducted to obtain the simple margins of predicted values by each social capital variables. This approach enables the calculation of the conditional effect of low household income on OHRQoL based on social capital variables, providing confidence intervals and p-values. A significance level of 0.05 was considered.

## Results

Out of the 558 pregnant women invited, 520 agreed to participate, yielding a 93% response rate. Among those who declined, the most commonly cited reasons were time constraints or lack of interest. Additionally, some participants tested positive for COVID-19, while a few expressed feelings of shame or fear.

The sample characteristics are described in Table 1. The pregnant women’s mean age was 26.9 (standard deviation [SD] 6.6). Most of them were white (60.2%) and presented high household income (74.4%). Regarding pregnancy time, 21% of women were in the first, 38% in the second, and 41% in the third trimester. Nearly 9 out of 10 participants reported having social support (92.9%), while a similar percentage did not attend prenatal groups (91.1%). Untreated dental caries was present in 38.3% of participants. The mean overall OHIP-14 score was 9.9 (SD 8.9).

**Table 1:** Demographic, socioeconomic, behavioral, psychosocial, and oral health characteristics of pregnant women (n = 520).

Variables	n (%)* (Mean- SD)
Demographic and socioeconomic	
Age	26.9 (6.6)
Skin color	
White	313 (60.2)
Black-brown	207 (39.8)
Household income in R$^†^	
High	387 (74.4)
Low	133 (25.6)
Pregnancy time (trimester)	
First	108 (21.0)
Second	194 (37.7)
Third	212 (41.3)
Behavior measure	
Frequency of dental attendance (time per year)	
> 1	280 (53.9)
< 1	240 (46.1)
Psychosocial measure – Social Capital	
Social support	
With	483 (92.9)
Without	37 (7.1)
Pregnant group attendance	
Yes	46 (8.9)
No	473 (91.1)
Oral health measures	
Untreated dental caries	
Present	321 (61.7)
Absent	199 (38.3)
Outcome	
OHIP-14	9.9 (8.9)

SD: standard deviation; OHIP: oral health impact profile. †Dichotomized according to the lowest quartile. *Values lower than 520 are indicate missing data.

Table 2 presents the multilevel unadjusted analysis, considering individual variables and the interaction between household income and social capital variables on overall OHIP-14 scores. In the unadjusted model, pregnant women with low household income and no social support exhibited poorer OHRQoL (p < 0.05). However, not attending a prenatal group was not associated with overall OHIP-14 scores (p = 0.274). In the interaction analysis, participants with low household income and no social support, as well as those with low household income who did not attend a prenatal group, were more likely to report poorer OHRQoL, with OHIP-14 rates 61% (RR = 1.61; 95%CI: 1.37–1.89) and 34% (RR = 1.34 95%CI: 1.16–1.56) higher, respectively, compared to their counterparts with high household income and high social capital.

**Table 2:** Unadjusted analysis of the interaction of household income, social support, and pregnant group attendance on overall OHIP-14 scores.

Variables	OHRQoL (OHIP-14)	
	RR (95%CI)	p-value
Household income in R$		
High	1 (reference)	< 0.001
Low	1.24 (1.15–1.35)	
Social support		
With	1 (reference)	< 0.001
Without	1.44 (1.31–1.58)	
Pregnant group attendance		
Yes	1 (reference)	0.274
No	1.06 (0.95–1.18)	
Interaction variables		
Household income x Social support		
High x With	1 (reference)	
High x Without	1.49 (1.32–1.67)	< 0.001
Low x With	1.24 (1.14–1.35)	< 0.001
Low x Without	1.61 (1.37–1.89)	< 0.001
Household income x Pregnant group attendance		
High x Yes	1 (reference)	0.261
High x No	1.07 (0.94–1.23)	0.043
Low x Yes	1.25 (1.00–1.57)	< 0.001
Low x No	1.34 (1.16–1.56)	

OHRQoL: oral health-related quality of life; OHIP: oral health impact profile; RR: rate ratio; CI: confidence interval; R$: Brazilian currency Real.

Adjusted analysis of the interaction of household income, social support, and pregnant group attendance on overall OHIP-14 scores are shown in Table 3. Pregnant women with low household income and no social support had overall OHIP-14 scores that were 44% higher (RR = 1.44; 95%CI: 1.22–1.72) compared to those with high household income and social support. Similarly, participants with low household income who did not attend a prenatal group experienced an approximately 22% greater impact on OHRQoL (RR = 1.22; 95%CI: 1.05–1.42). The absence of social support and lack of prenatal group attendance amplified the negative effects of low household income on OHRQoL. Additionally, high-income pregnant women without social support also experienced greater OHRQoL impacts compared to those with high income and adequate social support, while the reverse was true for low-income women with high social support) (p < 0.001).

**Table 3:** Adjusted analysis of the interaction of household income, social support, and pregnant group attendance on overall OHIP-14 scores.

Interaction variables	OHRQoL (OHIP–14)	
	RR (95% CI) *	p-value
Household income x Social support		
High x Yes	1 (reference)	
High x No	1.39 (1.23–1.57)	< 0.001
Low x Yes	1.16 (1.07–1.26)	< 0.001
Low x No	1.44 (1.22–1.72)	< 0.001
Household income x Pregnant group attendance		
High x Yes	1 (reference)	0.481
High x No	1.04 (0.91–1.19)	0.233
Low x Yes	1.14 (0.91–1.43)	0.009
Low x No	1.22 (1.05–1.42)	

OHRQoL: oral health-related quality of life; OHIP: oral health impact profile; RR: rate ratio; CI: confidence interval; *Adjusted for age, skin color, pregnancy trimester, frequency of dental attendance, and untreated dental caries.

Table 4 shows the predictive marginal effects of OHRQoL according to different dimensions of social capital among low-income pregnant women. The negative impact of low household income on OHRQoL was statistically significant across all social capital variables and levels (p < 0.001). The greatest margin effect was observed among low-income pregnant women without social support (13.35; 95%CI: 10.85–15.84).

**Table 4:** Predictive marginal effects of OHRQoL according to different social capital dimensions among pregnant women with low household income.

Interaction variables	OHRQoL	
	Margin (95%CI)	p-value
Low household income		
With Social support	10.69 (9.52-11.87)	< 0.001
Without Social support	13.35 (10.85-15.84)	< 0.001
Low household income		
Pregnant group attendance	9.43 (8.54-10.32)	< 0.001
No pregnant group attendance	11.00 (9.81-12.19)	< 0.001

CI: confidence interval.

## Discussion

This study aimed to evaluate the moderating effect of social capital on the relationship between household income and OHRQoL. Our findings are in agreement with the conceptual hypothesis that lower levels of social capital could intensify the impact of low household income on poor OHRQoL. Although previous studies have considered these factors, the moderating effect of social capital on a subjective oral health outcome in pregnant women has not been explored yet.

Pregnant women with a low household income presented poorer OHRQoL, in agreement with previous studies in different populations.^3^ We considered household income as a proxy for individual socioeconomic status.^6^ Individuals from lower socioeconomic backgrounds are more likely to be exposed to several risk factors for their health.^9,10^ Thus, these individuals have less access to dental services and worse health habits, which may lead to worse levels of oral health.^10,11^These individuals may also have greater psychosocial distress due to the environment in which they live, which also has repercussions on poorer levels of oral health and quality of life.

It is hypothesized that the impact of low income can be exacerbated during pregnancy, given that during this period, women undergo numerous social, emotional, and physical changes and adaptations.^23,24^Factors that can modify this relationship, such as social capital, may be relevant in this period.^33^ Our findings showed that pregnant women with both low household income and low social capital presented poorer OHRQoL, in agreement with previous studies that showed that social capital exerts positive effects on oral health,^34,35^ including among pregnant women.^16^ Individuals with high levels of social capital are more likely to suffer ‘peer pressure’ to have good health habits and use dental services, which may impact subjective oral health,^35^ irrespective of socioeconomic background. Higher social capital levels can also act as a protective factor against stress through feelings of security and belonging,^35^ positively impacting health. Analyzing the social capital variables separately, we found that social support had the greatest impact during pregnancy. Social capital can be divided into two dimensions — structural and cognitive.^13,14^ The structural dimension is primarily quantitative, focusing on the number and frequency of social networks, while the cognitive dimension is qualitative, reflecting how individuals perceive and feel about these networks.^13,14^ Previous studies in other populations have shown that the cognitive dimension tends to have a greater influence on subjective oral health.^20,30^ Although both dimensions are dynamically linked, the level of trust derived from these relationships appears to be essential for understanding the effects on individuals who rely on this resource. Although attending prenatal groups is important, the way a pregnant woman feels about her social connections is particularly critical for her well-being and overall quality of life.

Several theoretical frameworks can provide deeper insights into this finding. The absence of perceived social support from family members or friends can be especially detrimental during pregnancy, especially for women with low socioeconomic status, who are already exposed to multiple health risk factors.^10^ These women do not benefit from the protective effects of social support, which can mitigate the negative impacts of oral health problems on quality of life.^18^ Previous studies have also shown that low social support is associated with an increased risk of depression, anxiety, and self-harm during pregnancy,^33,36^as well as poorer OHRQoL.^16^ Additionally, the prevalence of prenatal group attendance was low in our sample (about 9%), which could partly explain why the structural dimension had a smaller role in the outcome. Low household income may restrict access to these specialized groups. Therefore, future studies are encouraged to investigate other indicators of social capital to further elucidate these findings.

This study has some limitations that should be acknowledged. Both social capital variables were evaluated using simple questions, which may limit the precision of this construct’s measurement. Nevertheless, social capital cannot be directly measured and is typically assessed through indicators or proxies.^13,37^ Additionally, socioeconomic status was measured solely by household income. Although this indicator is one of the most widely used in oral health research^6^ and has been identified as having the greatest negative effect on OHRQoL across various age groups,^3^ relying on a single measure may not capture the full complexity of socioeconomic status. Finally, due to the inherent limitations of cross-sectional designs, future studies are encouraged to investigate the causal pathways and interrelationships among socioeconomic status, social capital, and OHRQoL more comprehensively.

Despite its limitations, this study also has notable strengths. First, it involved a representative sample of pregnant women from southern Brazil, which enhances the external validity of our findings. Second, it examined key determinants and modifiers of OHRQoL during a critical period in many women’s lives. While low socioeconomic status exerts a significant negative influence on oral health, strategies aimed at fostering different forms of social capital may offer promising ways for improving oral health in this population.

## Conclusion

Our findings highlight that the absence of diverse forms of social capital may exacerbate the negative effects of low household income on OHRQoL among pregnant women. Additionally, our results indicate that social support plays a somewhat more significant role in moderating these effects. Future studies that test group-based prenatal programs, community and family involvement, digital support platforms, and mentorship initiatives for strengthening social connections among pregnant women should be encouraged. By strengthening social capital, healthcare services can help mitigate the negative effects of low socioeconomic status on OHRQoL.

## Data Availability

The datasets generated during and/or analyzed during the current study are available from the corresponding author on reasonable request.
